# Simple structured hybrid WOLEDs based on incomplete energy transfer mechanism: from blue exciplex to orange dopant

**DOI:** 10.1038/srep10234

**Published:** 2015-05-15

**Authors:** Tianyou Zhang, Bo Zhao, Bei Chu, Wenlian Li, Zisheng Su, Xingwu Yan, Chengyuan Liu, Hairuo Wu, Yuan Gao, Fangming Jin, Fuhua Hou

**Affiliations:** 1 State Key Laboratory of Luminescence and application, Changchun Institute of Optics, Fine Mechanics and Physics, Chinese Academy of Sciences, 3888-Dong NanHu Road, Changchun 130033, P. R. China; 2 Graduate School of the Chinese Academy of Sciences, Beijing 100039, P.R. China

## Abstract

Exciplex is well known as a charge transfer state formed between electron-donating and electron-accepting molecules. However, exciplex based organic light emitting diodes (OLED) often performed low efficiencies relative to pure phosphorescent OLED and could hardly be used to construct white OLED (WOLED). In this work, a new mechanism is developed to realize efficient WOLED with extremely simple structure by redistributing the energy of triplet exciplex to both singlet exciplex and the orange dopant. The micro process of energy transfer could be directly examined by detailed photoluminescence decay measurement and time resolved photoluminescence analysis. This strategy overcomes the low reverse intersystem crossing efficiency of blue exciplex and complicated device structure of traditional WOLED, enables us to achieve efficient hybrid WOLEDs. Based on this mechanism, we have successfully constructed both exciplex-fluorescence and exciplex-phosphorescence hybrid WOLEDs with remarkable efficiencies.

Since the first white organic light-emitting diode (WOLED) was reported by the group of Kido^1^,^2^, tremendous academic, technical and industrial interest have been devoted to the improvement of the electroluminescence (EL) efficiency, color tunability, and long term stability of WOLEDs. With their light-weight, fast-aa, wide-view-angle, low driving voltage, high brightness and efficiency, large area, WOLEDs are anticipated as one of the next generation energy-saving and eco-friendly light sources^3^,^4^,^5^. Over the past decades, many efficient materials and ingenious device structures have been developed to improve the device efficiency and color quality of WOLED^6^,^7^. Up to now, two kinds of luminescence materials, fluorophor and phosphor, have been being mostly used to fabricate WOLED in three or two primary color structures with various functional layers^8^,^9^. Among these device structures, fluorescence-phosphorescence (f-p) hybrid WOLEDs attracts a lot of attentions because the quality and stability of blue phosphors are still unsatisfied^10^,^11^,^12^. On the other hand, the power efficacy of WOLED must high enough to replace the prevailing poor color qualitied fluorescent tubes^3^ with typical power efficiency (PE) up to 90 lmW^−1^. The hybrid WOLEDs, especially the blue-orange two primary color WOLEDs, with low turn on bias and simple device structure, have the greatest potential to achieve high PE^13^,^14^.

Recently, in addition to the traditional materials, a kind of efficient thermally activated delayed fluorescence (TADF) material emerged^15^,^16^, including exciplex and intra-molecular charge transferred materials^17^,^18^,^19^,^20^,^21^. Hereafter, works using TADF materials to fabricate WOLED were also gradually carried out^14^,^22^,^23^. However, these works were limited to a simple combinational stage and some advantages of the TADF materials, especially the exciplexes, still not paid sufficient attention^24^. On the other hand, the exciplexes have been recently employed as the host of phosphors to achieve monochromatic devices with high efficiency and low turn bias^25^,^26^,^27^,^28^. But these strategies have only paid a little attention in fabricating WOLEDs^29^.

In this work, using an efficient blue exciplex^24^, we demonstrated that the energy could transfer from blue exciplex to both fluorescent and phosphorescent orange dopant. What’s more, we explored a new strategy to realize efficient WOLED with simple structures by making efficient use of the triplet exciplex. Triplet harvesting is a promising concept for future high-efficiency white OLEDs with high color quality. Once the right materials are found it will also allow us to simplify the device structure, because in general all materials could be blended into one uniform emission layer^3^.

For the exciplex OLED, the internal electroluminescence (EL) efficiency (ϕ_EL(int)_) can be calculated as follows^30^:
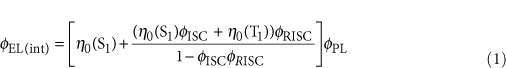

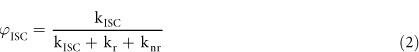

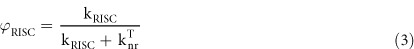
where *η*_0_(S_1_) and *η*_0_(T_1_) are the branching ratio of singlet and triplet exciplex formation (0.25 and 0.75), *ϕ*_RISC_ is the efficiency of reverse intersystem crossing (RISC), and *ϕ*_PL_ is the photoluminescence (PL) efficiency of exciplex. *ϕ*_ISC_ is the efficiency of ISC, k_ISC_ and k_RISC_ are the intersystem crossing (ISC) and the RISC rate, respectively. k_nr,T_ is the total non-radiative rate for the triplet exciplex decay processes except RISC, k_r_ and k_nr_ are the radiative and non-radiative decay rate of the singlet exciplex. When the exciplex emission is efficient, and k_r_»k_ISC_ and k_nr_, the efficiency of exciplex would mainly be limited by k_RISC_. The internal EL efficiency can be simplified as:
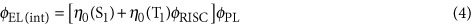


When a small concentration of orange dopant is present, the energy in triplet exciplex would be redistributed and could incompletely transfer from triplet exciplex states to the dopant with high PL efficiency (*ϕ*_PLD_). In this kind of hybrid WOLEDs, the internal EL efficiency (*ϕ*_H(int)_) could be present as:
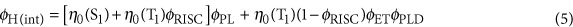
where *ϕ*_ET_ is the efficiency of energy transfer from triplet exciplex to the orange phosphor. Because the triplet exciplexes have to up converted to the singlet level in a relative long time scale, only part of the triplet exciplex in this study was finally converted to the delayed fluorescence, i.e., a *ϕ*_RISC_ is smaller than unity. The extra triplet exciplex would convert to heat and harm the device via non-radiative recombination route. When a small concentration dopant was doped into the exciplex or a near layer with modest dopant concentration was given, the extra triplet could be made full use of and WOLED with high EQE of above 20% could be achieved. Using this method, our WOLED performed a very low turn on bias of ca. 2.5 V and a high PE of 52.28 lmW^−1^. On the other hand, when doped with traditional fluorescent yellow dopant, efficient WOLED could still be achieved. But the EQE of the exciplex-fluorescence based hybrid WOLED was limited by the original blue exciplex because the energy could only transfer from singlet exciplex state to the fluorescent dopant. However, this strategy still paves the way to the low cost aim of large scale produce if the blue exciplex’s EQE can be enhanced to 20% in future.

We firstly fabricated the blue exciplex device using 9,9’-biphenyl-3, 3’-diylbis-9H -carbazole (mCBP) and PO-T2T as electron donor and acceptor components, respectively^24^. The molecular structures of mCBP^31^, PO-T2T and all orange dopants used in this work are shown in Fig. S1. The device structure was simple because mCBP and PO-T2T was used as hole and electron transporting materials respectively at the same time (Fig. 1). None the less, a high EQE of 7.66% with a modest highest brightness of 5016 cdm^−2^ could still be realized. As reported by Wen-Yi Hung *et al.*^24^, the exciplex emission and PL decay character were quite the same as the mixed film using N,N-8-dicarbazolyl-3,5-benzene (mCP) as electron donor, as shown in Fig. 2a. To study the origin of high EQE of this blue exciplex, PL decays under a wide range of temperature from 16 K to 290 K were determined, as shown in Fig. S2a. The PL decays contain two components, a prompt and a delayed one, even at very low temperature of 16 K. According to the TADF theory of Adachi *et al.*, the long decay component was attributed to the RISC of triplet exciplexes because the two components shared the same spectrum (as shown in Fig. S2b). However, the RISC process need no further thermal activation because the energy gap between the singlet and triplet exciplex is almost zero^19^. The PL quantum yield (PLQY) of the mCBP:PO-T2T 50 mol% mixed film (thickness of 100 nm) was 34 ± 4% at room temperature. Supposing an out-coupling efficiency of 20%-30%, the maximal EQE of 7.66% indicates that the singlet yield in the device was 75.1%-112.6%. This result suggests that the measured PLQY was often under estimated as discuss in ref. 19. The under estimation of the PLQY are attributed to the additional charge transfer process between the excited donor/acceptor to the acceptor/donor in ground state, causing an inaccurate deduction of the *ϕ*_RISC_ from the device EQE. However, from comparison of PL decays at low temperature and room temperature, we found the lifetime of the delayed component was fiercely quenched by phonon from 23.8 μs to 7.0 μs. The relative PL intensity at 16 K was about 2.5 times of that at room temperature. These phenomena indicated that only a small amount of triplet exciplex formed as a result of spin statistics in EL process could convert into light via RISC process. The wasted triplet exciplex can be made full use of by introducing a small doses of orange phosphor dopant or a nearby phosphor doped layer to achieve high efficiency WOLED. As shown in Fig. 2a, by adding a 2 nm thickness of bis(2-phenyl-1,3-benzothiozolato-N,C2’) iridium (acetylacetonate) (Ir(bt)_2_(acac)) doped mCBP:PO-T2T layer,^32^ an excellent warm WOLED with Commission Internationale de L’Eclairage (CIE) coordinates of (0.418, 0.433) was demonstrated. As the operating bias increasing from 3 V to 11 V, the CIE coordinates of this device changed with only a small variance of α(±0.002, ±0.001), as displayed in Fig. 2e and listed Table 1. This WOLED performed a highest EQE of 22.21% and remained higher than 20% at a high brightness of 1253 cdm^-2^ as shown in Fig. 2c. The device structure was simple because both the donor and acceptor components possess high triplet exciton levels than the triplet exciplex level. On the other hand, since there is a large overlap between the blue exciplex emission and the absorption spectra of orange dopants, as indicated in Fig. 2f, the blue exciplex with high excited energy level could directly transfer its energy to both the phosphorescent and fluorescent orange dopant. Hence no additional host materials and exciton blocking layer were needed in the WOLED. In the end, a high PE of 52.28 lmW^−1^ was achieved. To the best of our knowledge, this is among the most efficient f-p hybrid WOLEDs^5^,^14^. Besides, the turn on bias of this device was as low as 2.5 V and less than 4 V at a brightness of 1 cdm^−2^ and 1000 cdm ^2^. We further simplified the WOLED device structure by doping a low concentration of Ir(bt)_2_(acac) of ca. 0.5 wt.% into the whole emitting layer to construct an one emitting layer (EML) based WOLED. The one EML based WOLED performed an EQE and PE of 17.06% and 39.89 lmW^−1^ respectively with a maximum brightness of near 40000 cdm^−2^. The EQE of the one EML based WOLED was lower than the two EML based WOLED, suggesting that a competition exist between blue exciplex and the phosphor dopant. As shown in Fig. 2b, the PL lifetime of blue exciplex located at ca. 473 nm was partly quenched by the phosphor dopant from ca. 2.3 μs to ca. 0.8 μs when 0.5 wt.% Ir(bt)_2_(acac) exist (all PL decay time analysis in this work are listed in the table S1). Meanwhile the 590 nm Ir(bt)_2_(acac) peak showed a longer PL lifetime of ca. 2.2 μs than its intrinsic PL decay time of ca. 0.8 μs (Figure S3c), indicating that an energy transfer process is exist between triplet exciplex and the phosphor dopant. The conclusion can be supported by the fact that the orange peak in the time resolved PL spectra lasted in a range of 1-50 μs, as shown in Fig. S2b. However, in the two EML based WOLED, only extra long-lived triplet exciplex reached the interface of the two EML could transfer its energy to the phosphor dopant. Kim *et al.* found that in the phosphor doped exciplex system the electron and hole recombined in the langevin recombination manner^33^. In this work, we should point out that the direct recombination process on the phosphor also played an important role for the high EQE (22.21%) of the bi-layered device because the one layer WOLED gave a relative low EQE (17.06%). On the other hand, the pure orange phosphorescent OLED with direct electron-hole recombination on the phosphor gave low EQE (15.53%) relative to the WOLEDs, which further confirmed that the energy transfer process contributed to the high EQE in WOLED.

In order to further probe into the energy transfer process from exciplex to fluorescent dopant, 5, 6, 11, 12-tetraphenylnaphthacene (Rubrene) was selected as the fluorescent dopant. We first fabricated orange device using mCBP:PO-T2T exciplex as the host. When the concentration of Rubrene was as large as 1.0 wt.%, the doped device showed no exciplex emitting in the EL spectra as displayed in Fig. 3a. In addition, the orange device performed a low EQE of only 3.9% with small efficiency roll off, implies a short life time of the emission, suggesting that there is no energy transferred from exciplex to the orange dopant (only trap effect exist). However, as the dose of dopant was decreased to ca. 0.4 wt.%, the doped device performed warm WOLED with a CIE coordinates of (0.3161, 0.4269) at a luminance of ca.1000 cdm^2^. In this case, the internal EL efficiency (*ϕ*_H(int)_) can be present as:
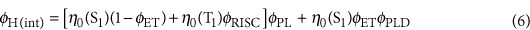
Where *ϕ*_ET_ is the efficiency of energy transfer from singlet exciplex to the orange fluorophor. The energy transfer process from singlet exciplex to the flourophor was determined by the PL decay and time resolved PL spectra analysis. The time resolved PL spectra of the 0.4 wt.% Rubrene doped mCBP:PO-T2T film was also examined, as shown in Fig. 3d. The fluorescent orange PL emission at 560 nm kept 50 μs and accompanied by the exciplex PL emission at ca. 473 nm. This long-lived fluorescence of Rubrene might also be originated from triplet-triplet annihilation (TTA) process because the Rubrene is a typical TTA material^34^. To ascertain the fact, we measured the PL decay of both pure Rubrene film and 1.0 wt.% Rubrene doped PO-T2T film. The PL lifetimes were extremely short in both films as shown in Fig. S3a and b, which was in consistent with previous reports^34^,^35^. However, the PL lifetime of the orange part in the WOLED became longer than 1 μs, as shown in Fig. 3c, further confirming the energy transfer process between singlet exciplex and the fluorescent dopant. Some may argue that the TTA process might be significant in the EL condition since 75% of excitons are formed as triplet states as a result of spin statistics. However, the pure orange Rubrene based device performed a small EQE of 3.9% which is far smaller than the EQE of the Rubrene based WOLEDs, suggesting that the energy transfer process contributed to the high EQE of the WOLEDs. To the best of our knowledge, this is the first time that a systematic study on energy transfer process between exciplex and fluorescent orange dopant is reported. It should also be noted that the energy transfer process was obviously only if small concentration of fluorophor was presented in this work. The WOLED consists of a 0.4 wt.% Rubrene doped mCBP:PO-T2T film performed an EQE and a PE of 6.09% and 9.16 lmW^−1^ respectively with a maximal brightness of 10005 cdm^−2^. In Fig. 3c, the PL decay time of the exciplex PL emission at ca. 473 nm also quenched to a short scale compared with that of the PL emission of non-doped mCBP:PO-T2T film. This implies that the energy transfer process from singlet exciplex to the orange dopant certainly reduce the emission of blue exciplex. To make a better trade off, we fabricated a two EML device comprised of complementary colors of blue exciplex and fluorescent orange emission. A better device performance of an EQE and PE of 7.05% and 11.44 l mW^−1^ was achieved. Both the one EML and two EML WOLED had a turn on voltage of less than 3 V. The EQE of 7.05% was higher than 5% but lower than 7.66%, implying that the WOLED using incomplete energy transfer from blue singlet exciplex to orange fluorescent dopant is efficient but limited by the efficiency of the blue exciplex. This result paves the way to high efficiency all-fluorescence WOLED with simple structure when the EQE of blue exciplex can be enhanced to near 20%.

It is interesting that the EL spectra of orange dopant in WOLED blue-shifted slightly and consequently damaged the quality of the WOLED, as shown in Fig. 3a. This disadvantage of the doped one EML based device could be improved by replacing Rubrene with the red dopant of 4-(dicyanomethylene)-2-t-butyl-6-(1,1,7,7-tetramethyljulolidyl-9-enyl)-4H-pyran (DCJTB). As shown in Fig. 4a, the peak position of DCJTB could be manipulated by controlling the dose in the mixed mCBP:PO-T2T film. When the concentration of DCJTB was decreased from ca.0.4 wt.% to ca.0.2 wt.%, the peak position of its EL spectra blue-shifted from ca. 600 nm to ca. 590 nm accordingly, which was far from its pristine EL spectra with peak position of 624 nm^36^. Using this character of fluorescent dopant, we successfully fabricated one EML based warm WOLED and cold WOLED with concentration of DCJTB of ca.0.4 wt.% and ca.0.2 wt.%. High EQEs of 6.16% and 5.75% were obtained in the two WOLED with a small turn on bias of less than 3 V (Fig. 4b). The warm DCJTB based WOLED performed high PE of 10.39 lmW^-1^ and a steady CIE coordinates of (0.452, 0.393) with insignificant variance of (±0.001, ±0.001) in a wide bias range of 4-12 V (Figs. 4c,4d). The qualities, luminance and efficiencies of the one EML Rubrene and DCJTB based WOLEDs were comparable to those sophisticated all fluorescent WOLEDs^3^.

We also combined the efficient blue exciplex with 4CzTPN-Ph, a famous orange colored TADF material, to fabricate WOLED^18^. It is interesting that the TADF material didn’t work well together with the blue exciplex due to complicated exciplex formation between the orange and the donor (acceptor) component when 4CzTPN-Ph was doped into the mCBP:PO-T2T mixed film (data not shown here). However, it didn’t prevent us from constructing WOLED with layer-by-layer structure. As shown in Fig. 4a, when an additional layer of PO-T2T:5.0 wt.% 4CzTPN-Ph was added into the exciplex device behind the exciplex layer, WOLED could still be realized with a modest EQE of 4.79% and a maximal luminance of 6781 cdm^−2^. The CIE coordinates of this device changed from (0.4806, 0.4479) to (0.3290, 0.3572) as the operating bias increased from 4 V to 11 V, as shown in Fig. 4d. No incomplete energy transfer process from triplet exciplex to the TADF dopant was observed, suggesting that the intra molecular charge transfer materials are more suit for one component host structure.

In summary, we have successfully explored a new strategy to realize simple structured highly efficient WOLED based on redistribution of the energy of blue exciplex even the efficiency of blue exciplex was limited compared with 20%. When doped with small concentration of orange phosphor or an adjacent orange phosphor doped layer was presented, the extra triplet exciplex could incompletely transfer its energy to the phosphor and finally gave efficient total white light emission. The device structures were extremely simple with only one or two EML. The turn-on biases were as low as 2.5 V and the efficiencies were comparable to those of all phosphorescent WOLEDs. When the exciplex was doped with fluorescent dopants, the energy could be transferred from the singlet exciplex to the orange dopant. The efficiencies of the WOLEDs with fluorescent dopants were high but limited by the efficiency of blue exciplex. However, this strategy still paves the way to high efficiency WOLED with fluorescent dopants when efficient blue exciplex is explored.

## Experimental Section

OLED devices were fabricated using pre-cleaned ITO-coated glass substrates with a sheet resistance of 15 Ω cm^−2^ and ITO thickness of 150 nm. They were patterned so that the OLED devices had a pixel size of about 12 mm^2^. The small molecule and cathode layers were thermally evaporated using the multiple-source organic molecule deposition method. The devices were prepared in vacuum at a pressure of 5*10^−4^ Pa. The MoO_3_, hole-transporting material mCBP, electron-transporting material PO-T2T were thermally evaporated at a rate of 0.1 nms^−1^. Blends of mCBP:PO-T2T and mCBP:PO-T2T:dopant were deposited at a rate of 0.1-0.2 nms^−1^. After the organic film deposition, a 0.8 nm layer of LiF and 150 nm layer of aluminum were thermally evaporated onto the organic surface. PL spectra of all the films were measured using Hitachi F7000 spectrometer. PL decay times and time resolved PL spectra of all the films were determined by FLS980 Fluorescence spectrometer. Current-voltage-brightness characteristics were measured by using a Keithley source measurement unit (Keithley2400) with a calibrated silicon photodiode. The EL spectra were measured by a Spectra scan PR650 spectrophotometer. All the EL measurements were carried out at room temperature under ambient condition.

## Author Contributions

**Figure 1:**
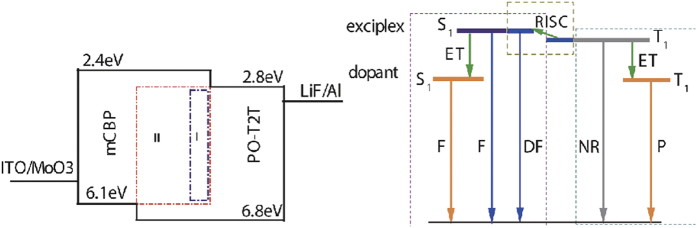
Device structure and schematic diagram of incomplete energy transfer from blue exciplex to the orange phosphor/fluorophor. Left: domain I and II in blue dash dot and red dash rectangles indicate the dope areas of orange dopant, correspond to WOLED with two emitting layer (EML) and one EML. Right: Jablonski diagram of incomplete energy transfer from exciplex to orange phosphor/fluorophor in the WOLED. F denotes fluorescence, DF denotes delayed fluorescence as a result of reverse intersystem crossing of triplet exciplex to the singlet exciplex state, and RISC denotes the reverse intersystem crossing process. ET denotes the part of triplet/singlet exciplex transfer its energy to the orange phosphor/fluorophor. NR denotes non-radiative process of triplet exciplex. P denotes phosphorescence from phosphor dopant.

**Figure 2:**
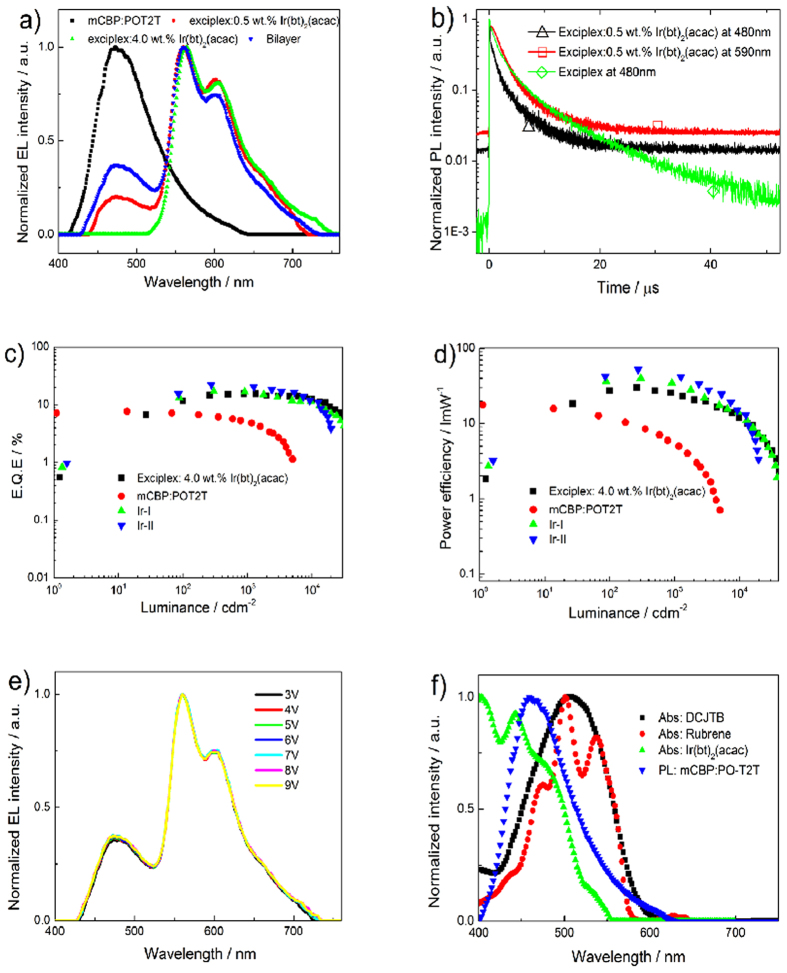
(**a**) Normalized EL spectra of mCBP:PO-T2T exciplex based device, Ir(bt)_2_(acac) doped one EML and two EML WOLED as well as the Ir(bt)_2_(acac) based pure orange Phosphorescent OLED. (**b**) PL decay characters of mCBP:PO-T2T mixed film and mCBP:PO-T2T:0.5 wt.% Ir(bt)_2_(acac) doped film. The excitation wavelength was 266 nm. (**c**) PE and (**d**) EQE vs luminance of four devices. The device structures are: Exciplex: ITO/MoO_3_ (3 nm)/ mCBP (20 nm)/mCBP:PO-T2T (20 nm)/ PO-T2T (40 nm)/LiF (0.8 nm)/Al; Ir-I: ITO/MoO_3_ (3 nm)/ mCBP (20 nm)/mCBP:PO-T2T:0.5 wt.% Ir(bt)_2_(acac) (20 nm)/ PO-T2T (40 nm)/LiF (0.8 nm)/Al, Ir-II: ITO/MoO_3_ (3 nm)/ mCBP (20 nm)/ mCBP:PO-T2T (20 nm)/ mCBP:PO-T2T:4.0 wt.% Ir(bt)_2_(acac) (2 nm)/ PO-T2T (40 nm)/LiF(0.8 nm)/Al; ITO/MoO_3_ (3 nm)/ mCBP (20 nm)/mCBP:PO-T2T:4.0 wt.% Ir(bt)_2_(acac) (20 nm)/ PO-T2T (40 nm)/LiF (0.8 nm)/Al. (**e**) EL spectra of Ir(bt)_2_(acac) doped two EML WOLED at different bias from 3 V to 9 V. (**f**) Normalized absorption spectra of Ir(bt)_2_(acac), Rubrene and DCJTB, with normalized PL spectra of mCBP:PO-T2T exciplex for comparison.

**Figure 3:**
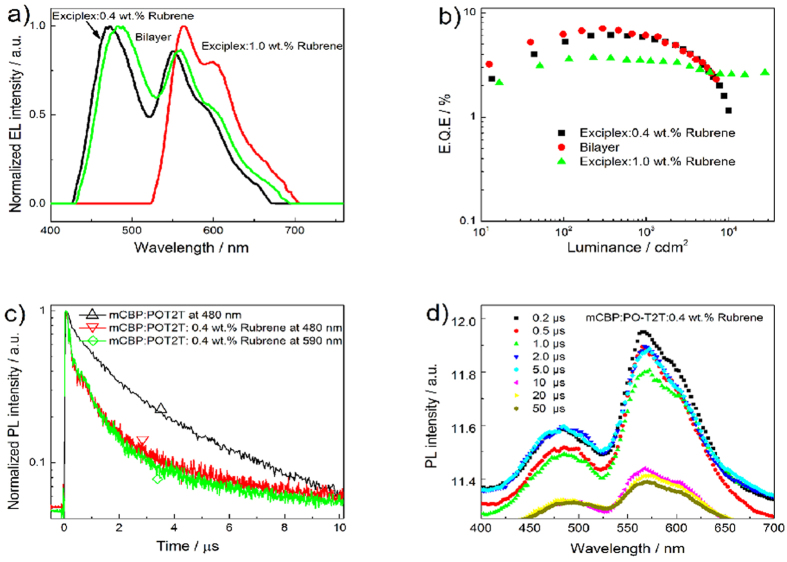
(**a**) Normalized EL spectra of Rubrene doped one EML and two EML WOLED as well as the Rubrene based pure orange OLED. (**b**) EQE vs brightness of three devices. The device structures are: Rubrene-I: ITO/MoO_3_ (3 nm)/mCBP (20 nm)/mCBP:PO-T2T:0.4 wt.% Rubrene (20 nm)/PO-T2T (40 nm)/LiF (0.8 nm)/Al; Rubrene-II: ITO/MoO_3_ (3 nm)/mCBP (20 nm)/mCBP:PO-T2T (20 nm)/mCBP:PO-T2T:1.0 wt.% Rubrene (5 nm)/PO-T2T (40 nm)/LiF (0.8 nm)/Al; ITO/MoO_3_ (3 nm)/mCBP (20 nm)/mCBP:PO-T2T:1.0 wt.% Rubrene (20 nm)/PO-T2T (40 nm)/LiF (0.8 nm)/Al. (**c**) PL decay characters of mCBP:PO-T2T mixed film and mCBP:PO-T2T:0.4 wt.% Rubrene doped film. (**d**) Time resolved spectra of mCBP:PO-T2T:0.4 wt.% Rubrene doped film, excited at 266 nm.

T.Z. carried out the OLED device fabrication, measurement, and data analysis as well as P.L. decay dynamics. W.L., B.C. and Z.S. provided the necessary consultations during the write-up of the present article. X.Y., C.L. and B.Z. prepared figs. 1, 2, 3. F.J., Y.G. and H.W. and F.H. prepared the supplementary information. All the authors discussed the results and contributed to the article.

## Additional Information

**How to cite this article**: Zhang, T. *et al.* Simple structured hybrid WOLEDs based on incomplete energy transfer mechanism: from blue exciplex to orange dopant. *Sci. Rep.* **5**,10234 doi: 10.1038/srep10234 (2015).w

## Supplementary Material

Supporting Information

## Figures and Tables

**Figure 4:**
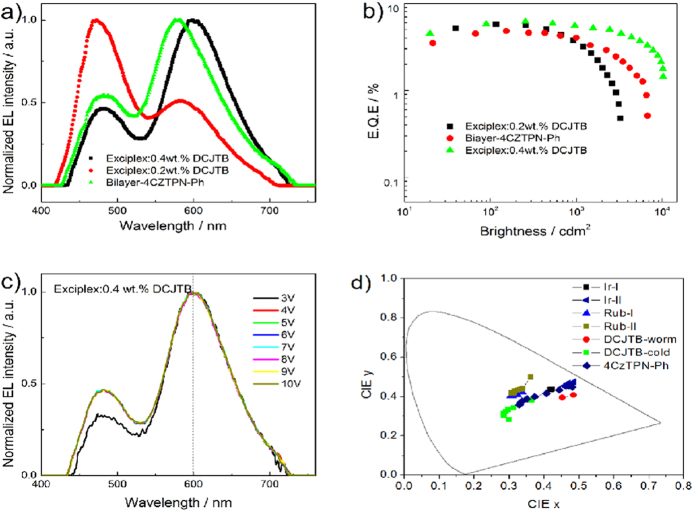
(**a**) Normalized EL spectra of DCJTB doped warm and cold WOLED as well as the 4CzTPN-Ph doped two EML WOLED. (**b**) EQE vs luminance of three devices. The device structures are: DCJTB-warm: ITO/MoO_3_ (3 nm)/mCBP (20 nm)/mCBP:PO-T2T:0.4 wt.% DCJTB (20 nm)/PO-T2T (40 nm)/LiF (0.8 nm)/Al, DCJTB-cold: ITO/MoO_3_ (3 nm)/mCBP (20 nm)/mCBP:PO-T2T:0.2 wt.% DCJTB (20 nm)/PO-T2T (40 nm)/LiF (0.8 nm)/Al, ITO/MoO_3_ (3 nm)/mCBP (20 nm)/mCBP:PO-T2T (20 nm)/PO-T2T:5.0 wt.% 4CzTPN-Ph (10 nm)/ PO-T2T (40 nm)/LiF (0.8 nm)/Al. (**c**) EL spectra of the DCJTB based warm WOLED at different bias from 3 V to 10 V. (**d**) CIE coordinates of all WOLED in this work, as listed in Table 1.

**Table 1:** Summary of device performances of the WOLEDs and exciplex based OLED.

**Devices**	**EQE**^a^)**(EQE**^b^)**) %**	**CE** a)**(CE**^b^)**) cd/A**	**PE** a)**(PE**^b^)**) lm/W**	**Maximal Brightness cd/m**^2^	**CIE**^c^)**(x, y)**	
				
Exciplex	7.66(5.04)	15.08(9.95)	17.78(5.42)	5016	0.170	0.230
Ir-I	22.21(20.56)	58.35(54.01)	52.28(43.21)	19765	0.418	0.433
Ir-II	17.06(16.51)	44.52(43.53)	39.89(33.25)	38153	0.463	0.457
Rub-I	7.05(6.07)	18.22(15.63)	11.44(7.58)	10091	0.309	0.405
Rub-II	6.09(5.77)	14.76(13.97)	9.16(6.63)	10005	0.316	0.426
DCJTB-warm	6.16(5.51)	13.25(11.86)	10.39(7.39)	10289	0.452	0.393
DCJTB-cold	5.75(3.61)	11.88(7.47)	9.33(3.85)	3273	0.290	0.323
4CzTPN-Ph	4.79(4.02)	11.21(8.72)	7.32(3.95)	6781	0.407	0.416

^a^Maximal values.

^b^Values at 1000 cd/m^2^.

^c^Values at 6 V bias.

## References

[b1] Kido J., Hongawa K., Okuyama K. & Nagai K. White light‐emitting organic electroluminescent devices using the poly(N‐vinylcarbazole) emitter layer doped with three fluorescent dyes. Appl. Phys. Lett. 64, 815–817 (1994).

[b2] Kido J., Shionoya H. & Nagai K. Single‐layer white light‐emitting organic electroluminescent devices based on dye‐dispersed poly(N‐vinylcarbazole). Appl. Phys. Lett. 67, 2281–2283 (1995).

[b3] Reineke S., Thomschke M., Lüssem B. & Leo K. White organic light-emitting diodes: Status and perspective. Rev. Mod. Phys. 85, 1245–1293 (2013).

[b4] Kamtekar K. T., Monkman A. P. & Bryce M. R. Recent Advances in White Organic Light‐Emitting Materials and Devices (WOLEDs). Adv. Mater. 22, 572–582 (2010). 20217752 10.1002/adma.200902148

[b5] Chen J., Zhao F. & Ma D. Hybrid white OLEDs with fluorophors and phosphors. Mater. Today 17, 175–183 (2014).

[b6] Baldo M. A. *et al.* Highly efficient phosphorescent emission from organic electroluminescent devices. Nature 395, 151–154 (1998).

[b7] Kawamura Y., Yanagida S. & Forrest S. R. Energy transfer in polymer electrophosphorescent light emitting devices with single and multiple doped luminescent layers. J. Appl. Phys. 92, 87–93 (2002).

[b8] Tsai Y.-C. & Jou J.-H. Long-lifetime, high-efficiency white organic light-emitting diodes with mixed host composing double emission layers. Appl. Phys. Lett. 89, 243521–243523 (2006).

[b9] Rosenow T. C. *et al.* Highly efficient white organic light-emitting diodes based on fluorescent blue emitters. J. Appl. Phys. 108, 113113–113117 (2010).

[b10] Reineke S. *et al.* White organic light-emitting diodes with fluorescent tube efficiency. Nature 459, 234–238 (2009). 19444212 10.1038/nature08003

[b11] Schwartz G., Fehse K., Pfeiffer M., Walzer K. & Leo K. Highly efficient white organic light emitting diodes comprising an interlayer to separate fluorescent and phosphorescent regions. Appl. Phys. Lett. 89, 083509–083511 (2006).

[b12] Su S. J., Gonmori E., Sasabe H. & Kido J. Highly efficient organic blue‐and white‐light‐emitting devices having a carrier‐and exciton‐confining structure for reduced efficiency roll‐off. Adv. Mater. 20, 4189–4194 (2008).

[b13] Schwartz G., Pfeiffer M., Reineke S., Walzer K. & Leo K. Harvesting Triplet Excitons from Fluorescent Blue Emitters in White Organic Light‐Emitting Diodes. Adv. Mater. 19, 3672–3676 (2007).

[b14] Zheng C. J. *et al.* Novel efficient blue fluorophors with small singlet-triplet splitting: hosts for highly efficient fluorescence and phosphorescence hybrid WOLEDs with simplified structure. Adv. Mater. 25, 2205–2211 (2013). 23417718 10.1002/adma.201204724

[b15] Goushi K. & Adachi C. Efficient organic light-emitting diodes through up-conversion from triplet to singlet excited states of exciplexes. Appl. Phys. Lett. 101, 023306–023310 (2012).

[b16] Goushi K., Yoshida K., Sato K. & Adachi C. Organic light-emitting diodes employing efficient reverse intersystem crossing for triplet-to-singlet state conversion. Nat. Photon . 6, 253–258 (2012).

[b17] Hung W.-Y. *et al.* Highly Efficient Bilayer Interface Exciplex For Yellow Organic Light-Emitting Diode. ACS appl. mater. interfaces 5, 6826–6831 (2013). 23848982 10.1021/am402032z

[b18] Uoyama H., Goushi K., Shizu K., Nomura H. & Adachi C. Highly efficient organic light-emitting diodes from delayed fluorescence. Nature 492, 234–239 (2012). 23235877 10.1038/nature11687

[b19] Zhang T. *et al.* Efficient triplet application in exciplex delayed fluorescence OLEDs using reverse intersystem crossing mechanism based on a ∆E_(S−T)_ around zero. ACS appl. mater. interfaces 6, 11907–11914 (2014). 24840782 10.1021/am501164s

[b20] Zhang Q. *et al.* Efficient blue organic light-emitting diodes employing thermally activated delayed fluorescence. Nat Photon 8, 326–332 (2014).

[b21] Tao Y. *et al.* Thermally Activated Delayed Fluorescence Materials Towards the Breakthrough of Organoelectronics. Adv Mater 26, 7931–7958 (2014). 25230116 10.1002/adma.201402532

[b22] Kim B. S., Yook K. S. & Lee J. Y. Above 20% external quantum efficiency in novel hybrid white organic light-emitting diodes having green thermally activated delayed fluorescent emitter. Sci. Rep . 4, 6019–6024 (2014). 25317855 10.1038/srep06019 PMC5377539

[b23] Nishide J., Nakanotani H., Hiraga Y. & Adachi C. High-efficiency white organic light-emitting diodes using thermally activated delayed fluorescence. Appl. Phys. Lett. 104, 233304–233308 (2014).

[b24] Hung W.-Y. *et al.* The First Tandem, All-exciplex-based WOLED. Sci. Rep. 4, 5161–5166 (2014). 24895098 10.1038/srep05161 PMC4044637

[b25] Kim B. S. & Lee J. Y. Engineering of Mixed Host for High External Quantum Efficiency above 25% in Green Thermally Activated Delayed Fluorescence Device. Adv. Funct. Mater. 24, 3970–3977 (2014).

[b26] Kim K. H., Moon C. K., Lee J. H., Kim S. Y. & Kim J. J. Highly Efficient Organic Light‐Emitting Diodes with Phosphorescent Emitters Having High Quantum Yield and Horizontal Orientation of Transition Dipole Moments. Adv. Mater. 26, 3844–3847 (2014). 24715684 10.1002/adma.201305733

[b27] Seino Y., Sasabe H., Pu Y. J. & Kido J. High‐Performance Blue Phosphorescent OLEDs Using Energy Transfer from Exciplex. Adv. Mater. 26, 1612–1616 (2014). 24452829 10.1002/adma.201304253

[b28] Seo S. *et al.* Exciplex-triplet energy transfer: A new method to achieve extremely efficient organic light-emitting diode with external quantum efficiency over 30% and drive voltage below 3 V. Japanese Journal of Applied Physics 53, 042102–042109 (2014).

[b29] Lee S., Shin H. & Kim J. J. High‐Efficiency Orange and Tandem White Organic Light‐Emitting Diodes Using Phosphorescent Dyes with Horizontally Oriented Emitting Dipoles. Adv. Mater. 26, 5864–5868 (2014). 24923483 10.1002/adma.201400330

[b30] Zhang D. *et al.* High-efficiency fluorescent organic light-emitting devices using sensitizing hosts with a small singlet-triplet exchange energy. Adv Mater 26, 5050–5054 (2014). 24944186 10.1002/adma.201401476

[b31] Gong S. *et al.* Simple CBP isomers with high triplet energies for highly efficient blue electrophosphorescence. J. Mater. Chem. 22, 2894–2899 (2012).

[b32] Fan C. & Yang C. Yellow/orange emissive heavy-metal complexes as phosphors in monochromatic and white organic light-emitting devices. Chemical Society reviews 43, 6439–6469 (2014). 24927103 10.1039/c4cs00110a

[b33] Lee J. H., Lee S., Yoo S. J., Kim K. H. & Kim J. J. Langevin and Trap‐Assisted Recombination in Phosphorescent Organic Light Emitting Diodes. Adv. Funct. Mater. 24, 4681–4688 (2014).

[b34] Li F. *et al.* Electrical and optical characteristics of red organic light-emitting diodes doped with two guest dyes. Synthetic Metals 139, 341–346 (2003).

[b35] Ma L. *et al.* Fluorescence from rubrene single crystals: Interplay of singlet fission and energy trapping. Phys. Rev. B 87, 201203(R)–201207(R) (2013).

[b36] Jarikov V. V., Klubek K. P., Liao L.-S. & Brown C. T. Operating lifetime recovery in organic light-emitting diodes having an azaaromatic hole-blocking/electron-transporting layer. J. Appl. Phys. 104, 074914–074918 (2008).

