# Supplementary material for: Simple structured hybrid WOLEDs based on incomplete energy transfer mechanism: from blue exciplex to orange dopant
Source: Sci Rep. 2015 May 15;5:10234. doi: 10.1038/srep10234 (PMC4432569; doi:10.1038/srep10234)
Supplement: Supporting Information [file srep10234-s1.doc]

Supporting Information

**Simple structured hybrid WOLEDs based on incomplete energy transfer mechanism: from blue exciplex to orange dopant**

*Tianyou Zhang^1,2^, Bo Zhao^1,2^, Bei Chu^1^, Wenlian Li^1,*^, Zisheng Su^1^, Xingwu Yan^1,2^,* *Chengyuan Liu^1,2^*, *Hairuo Wu^1,2^, Yuan Gao^1,2^, Fangming Jin^1,2^, and Fuhua Hou^1,2^*

^1^State Key Laboratory of Luminescence and application, Changchun Institute of Optics, Fine Mechanics and Physics, Chinese Academy of Sciences, 3888-Dong NanHu Road, Changchun 130033, P. R. China

^2^Graduate School of the Chinese Academy of Sciences, Beijing 100039, P.R. China

**Corresponding Authors**

*E-mail: [wllioel@aliyun.com](mailto:wllioel@aliyun.com)

1. Molecular structures:


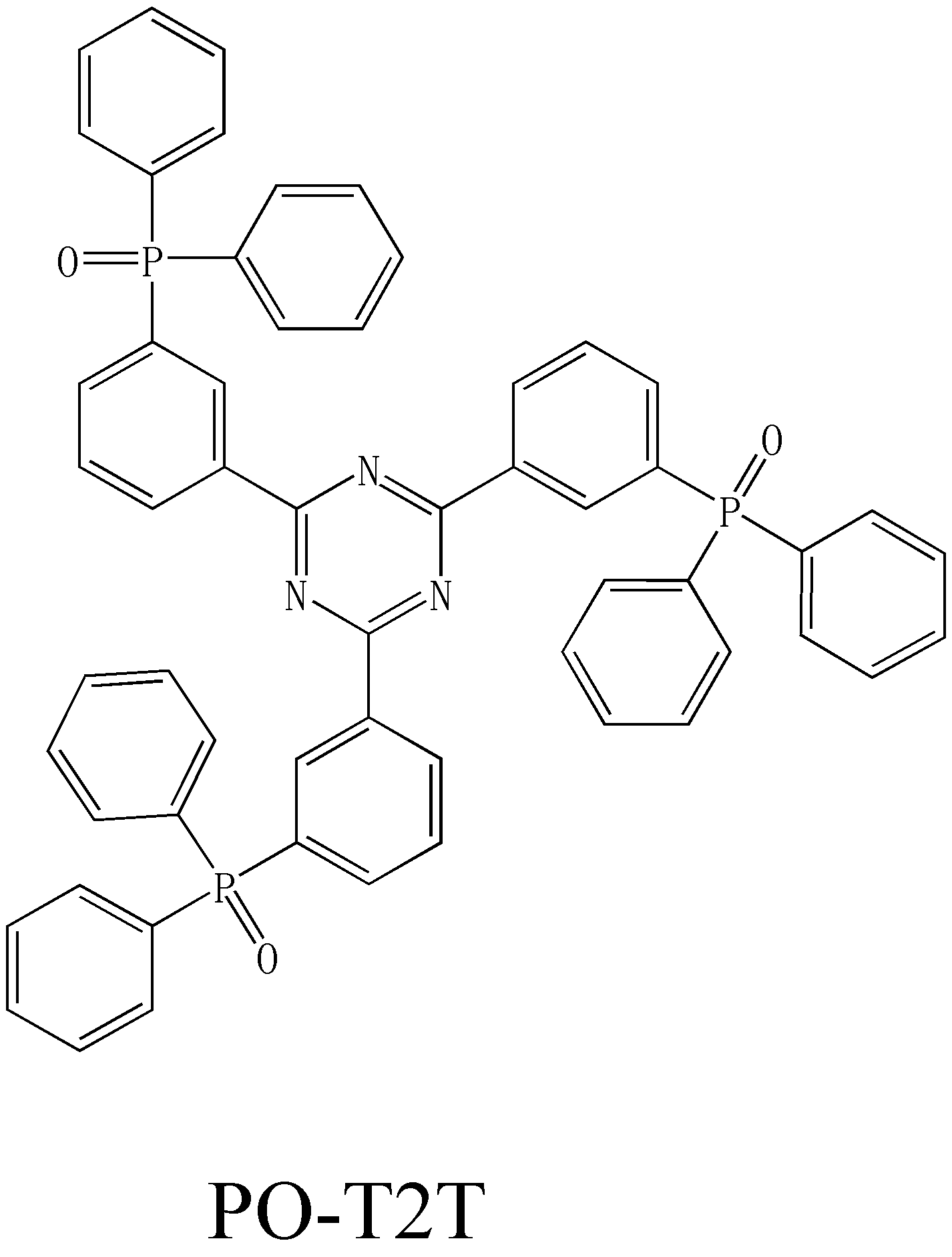

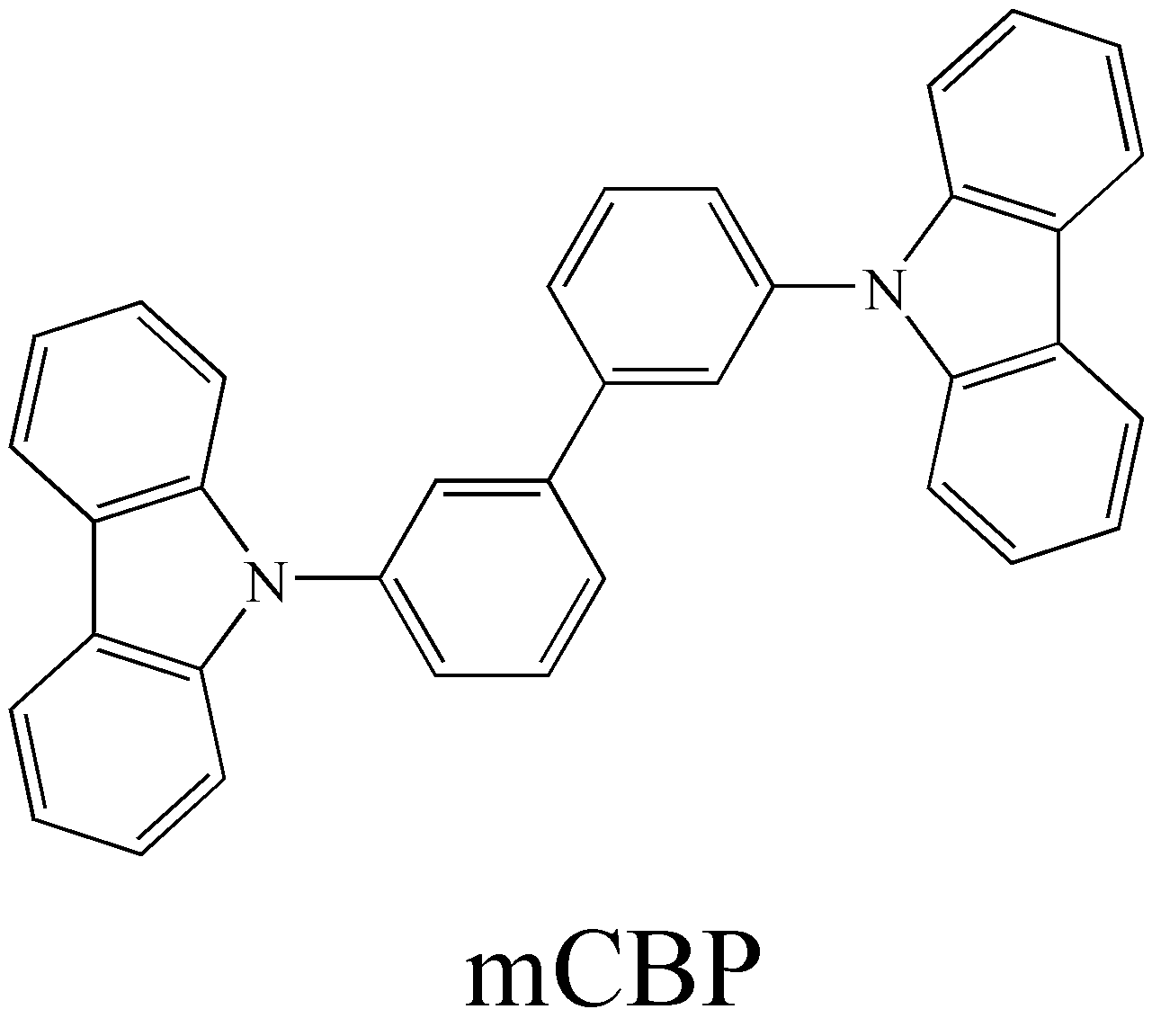


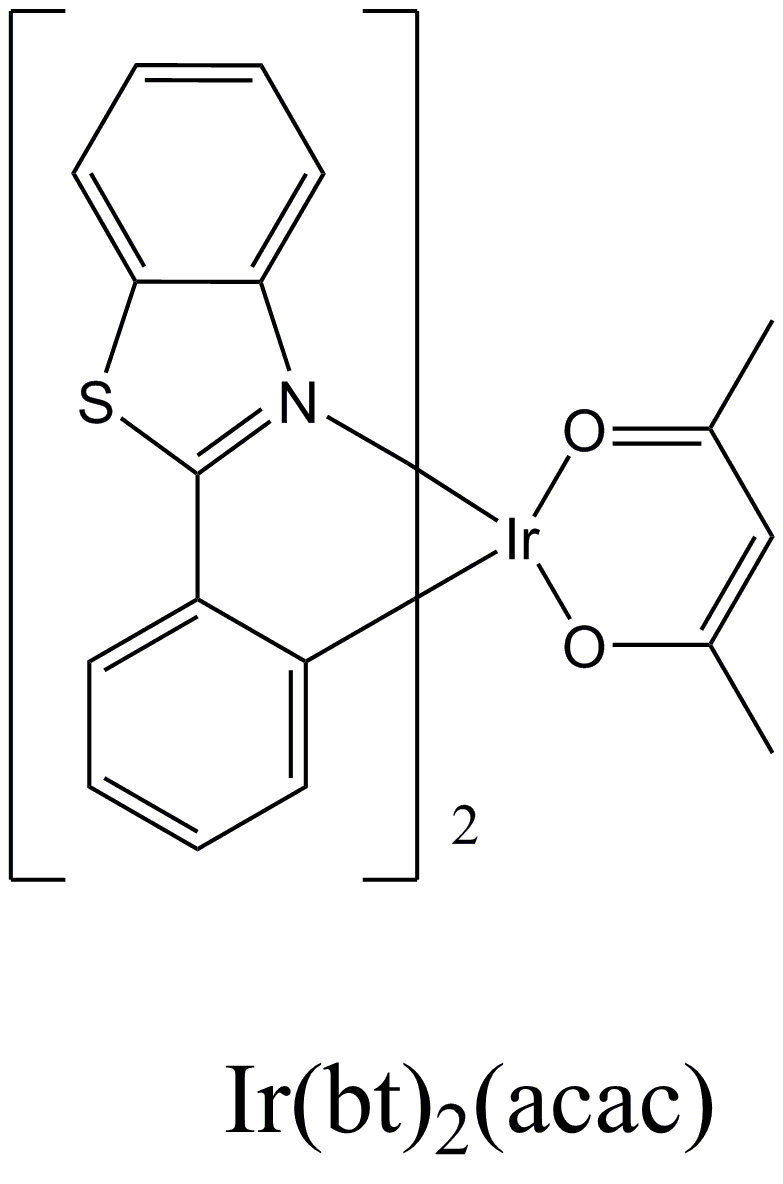

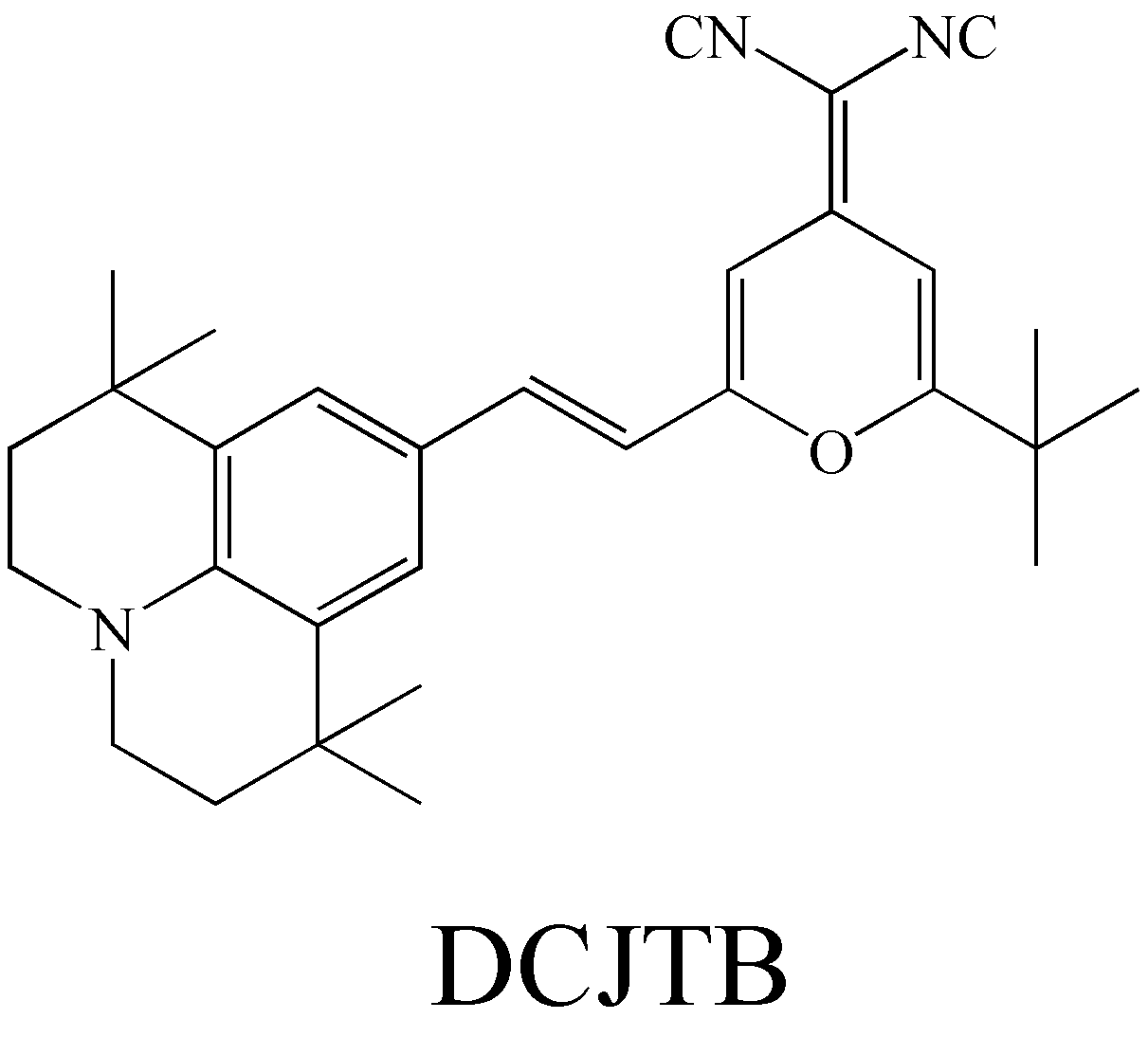

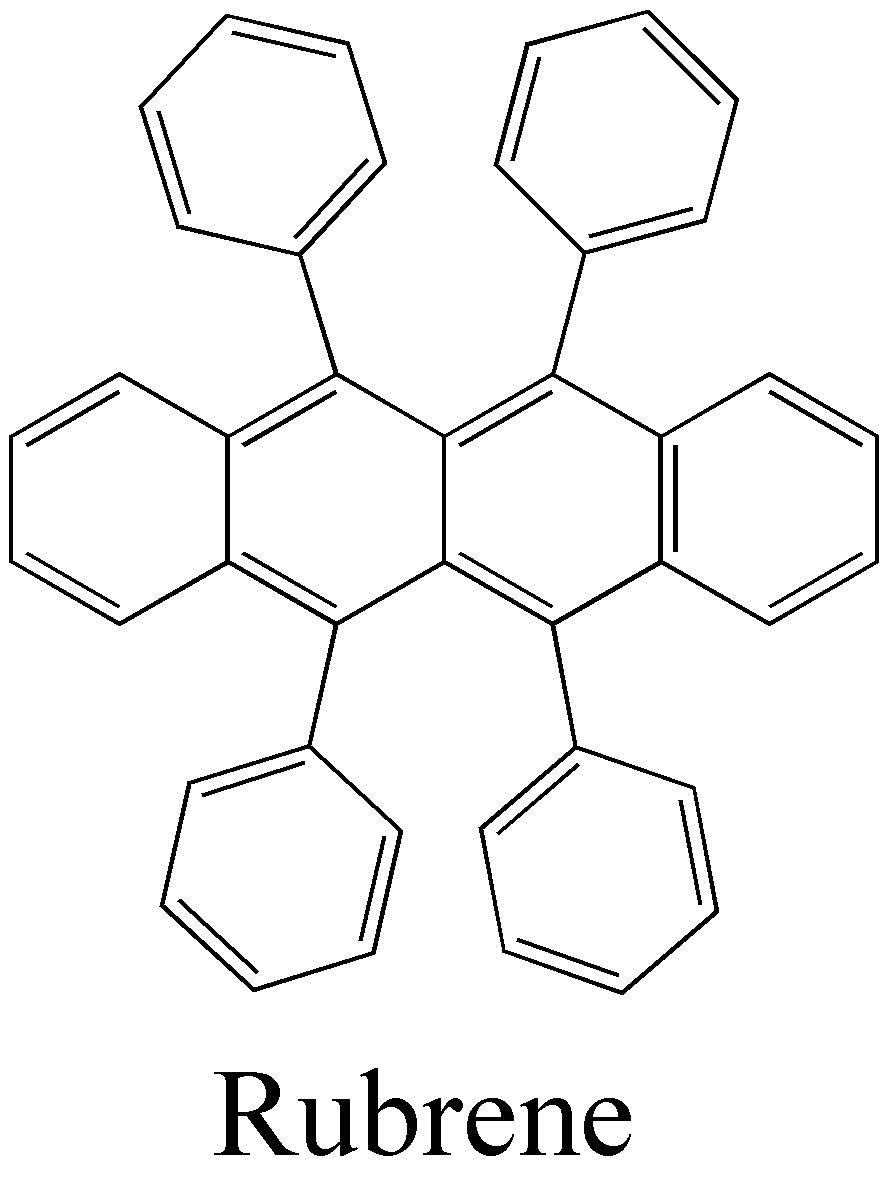


**Figure S1.** Molecular structures used in this work. PO-T2T was used as electron acceptor and electron transporting layer. mCBP was used as electron donor and hole transporting layer. Ir(bt)_2_(acac), Rubrene and DCJTB were used as phpsphorescent and fluorescent orange dopant.

2. PL decay characters:


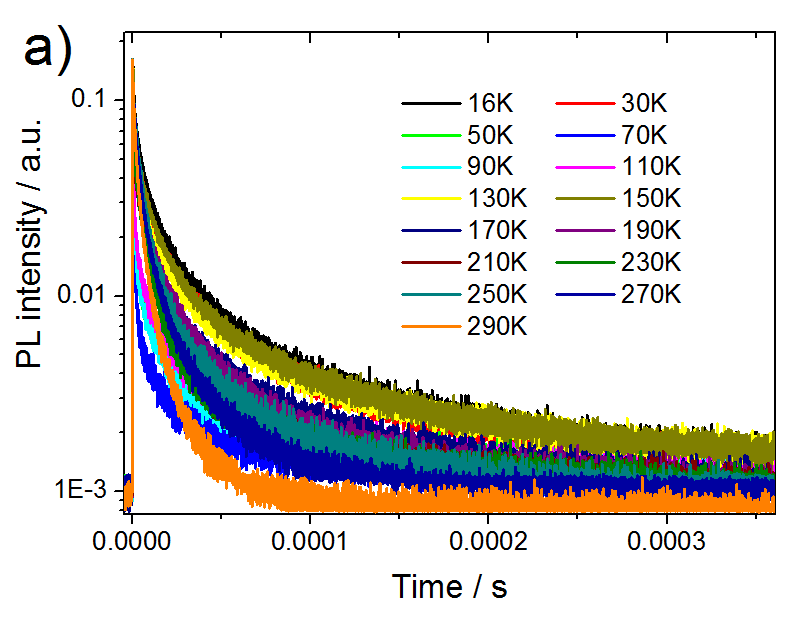

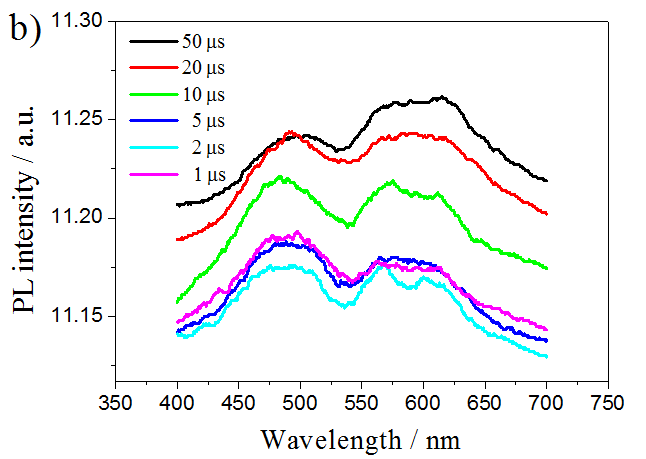


**Figure S2.** a) PL decay characters of mCBP:PO-T2T exciplex at different temperatures, monitored at 473 nm and excited at 266 nm. b)Time resolved spectra of mCBP:PO-T2T:0.5 wt.% Ir(bt)_2_(acac) film. The orange part from phosphor in the spetra tempal lasting to 50 μs and became stronger than the blue part from exciplex gave direct evidence of energy transfer from triplet exciplex to the phosphor.


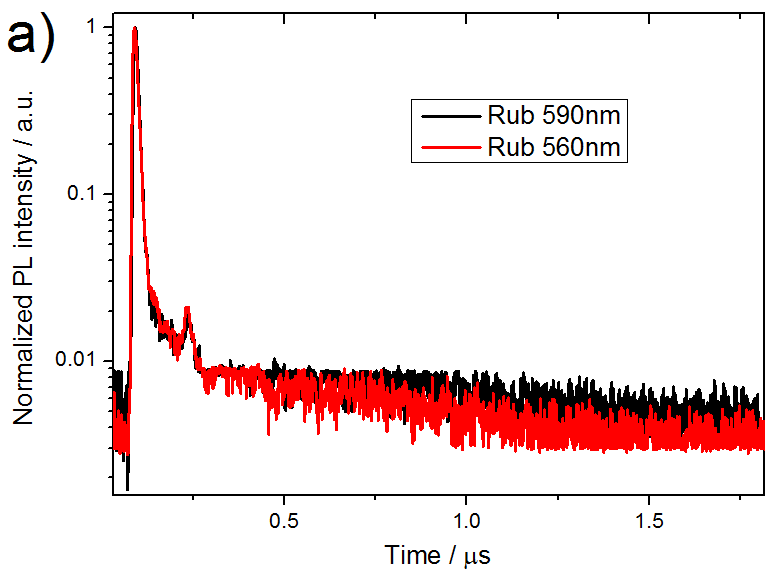

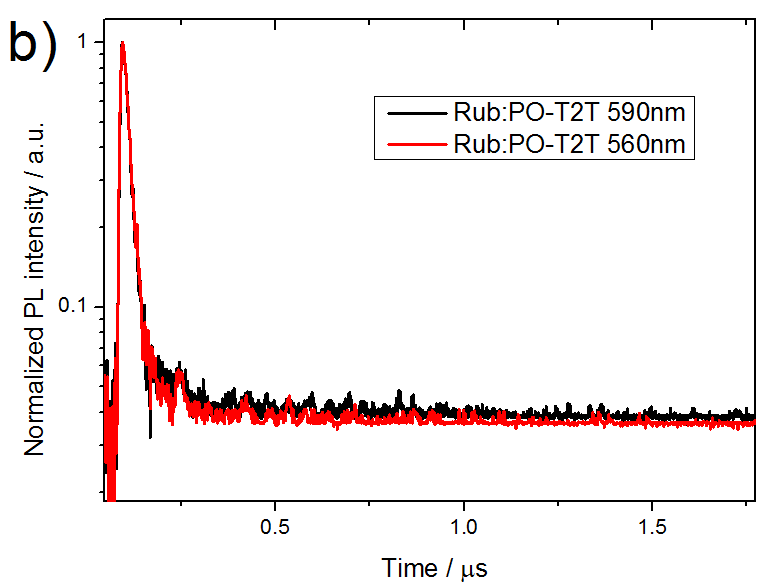

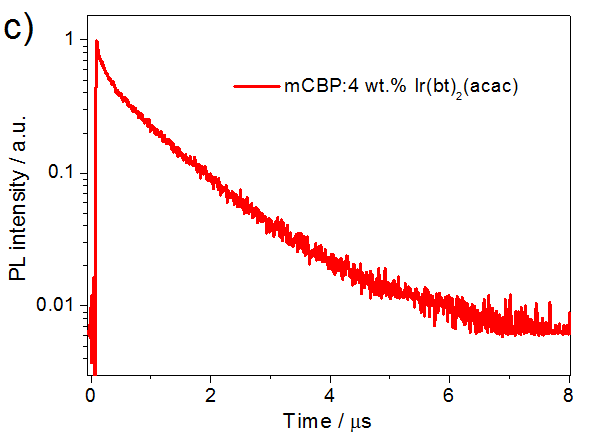


**Figure S3.** PL decay of a) pure Rubrene film, b) 1.0 wt.% Rubrene doped PO-T2T film and c) mCBP:4.0 wt.% Ir(bt)_2_(acac) film.

Table S1 decay characters of the doped and mixed films in this work.

| Films | Position  (nm) | Component1 | | Component2 | |
| --- | --- | --- | --- | --- | --- |
|  |  | Time/μs | Percentage | Time/μs | Percentage |
| mCBP:PO-T2T | 473 | 1.14 | 81% | 7.05 | 19% |
| mCBP:PO-T2T:0.5 wt.%Ir(bt)_2_(acac) | 473 | 0.15 | 70% | 2.25 | 30% |
| mCBP:PO-T2T:0.5 wt.%Ir(bt)_2_(acac) | 590 | 2.17 | 95% | 10.73 | 5% |
| mCBP:PO-T2T:0.4 wt.% Rubrene | 473 | 0.16 | 73% | 1.87 | 27% |
| mCBP:PO-T2T:0.4 wt.% Rubrene | 590 | 0.15 | 38% | 1.43 | 62% |
| mCBP:4.0 wt.% Ir(bt)_2_(acac) | 560 | 0.767 | 100% | - | - |

3. Device performance: luminance, current density, cuerrent efficiency and EL spectra.


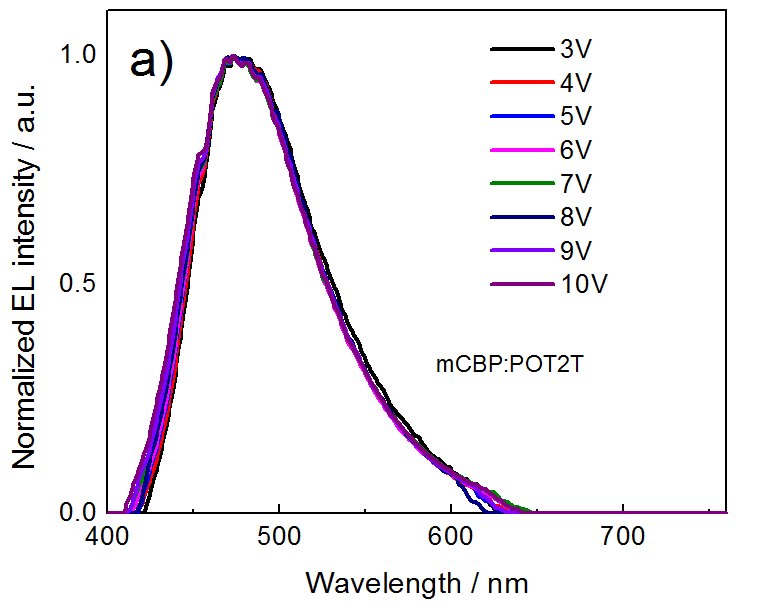

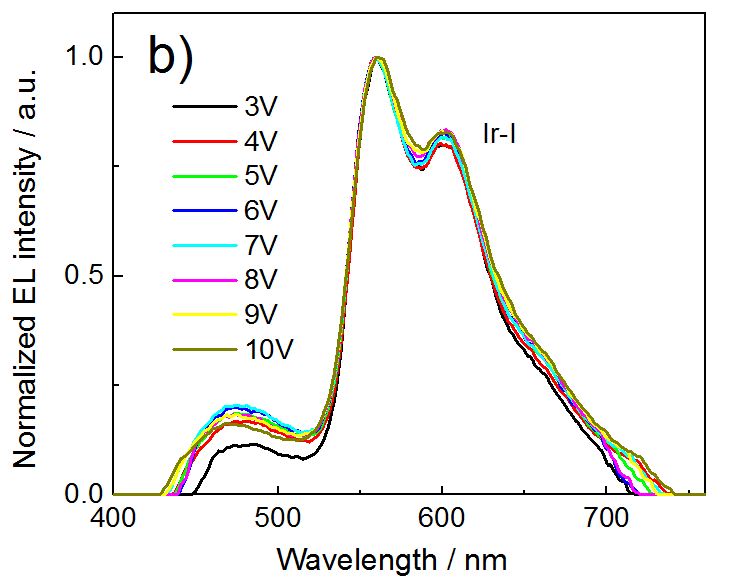


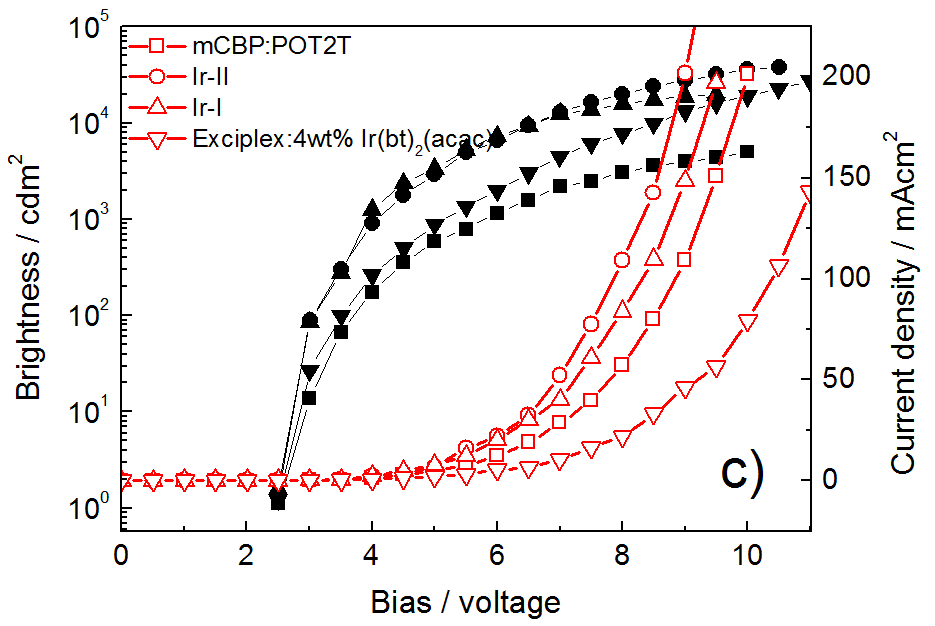

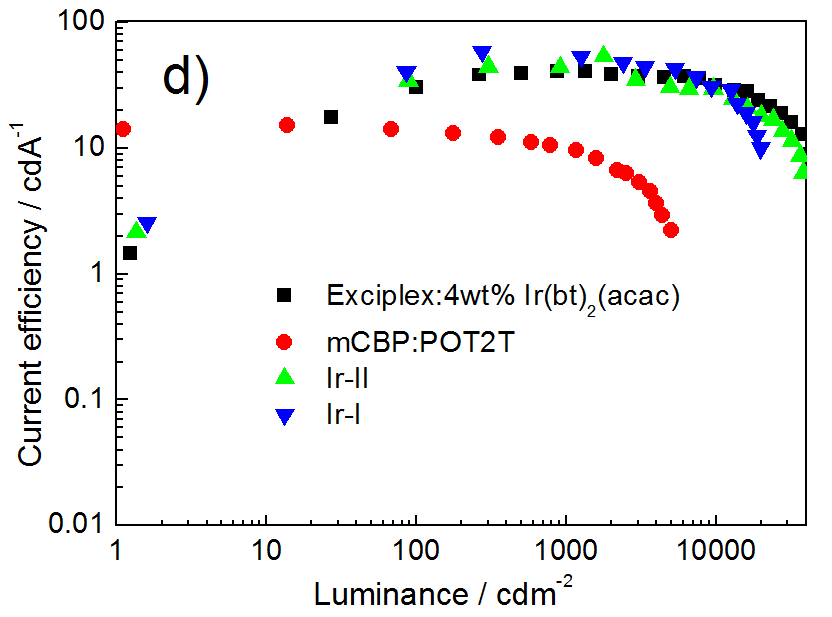


**Figure S4.** EL spectra of mCBP:PO-T2T exciplex based device a) and Ir(bt)_2_(acac) doped exciplex based one EML WOLED under bias from 3 V to 10 V. Luminance and current density c) and current efficiency d) of the exciplex based device, one EML and two EML Ir(bt)_2_(acac) based white devices and Ir(bt)_2_(acac) based orange device. The device structures are: ITO/MoO3 (3 nm)/ mCBP (20 nm)/mCBP:PO-T2T (20 nm)/ PO-T2T (40 nm)/LiF (0.8 nm)/Al; ITO/MoO3 (3 nm)/ mCBP (20 nm)/mCBP:PO-T2T:0.5 wt.% Ir(bt)_2_(acac) (20 nm)/ PO-T2T (40 nm)/LiF (0.8 nm)/Al; ITO/MoO3 (3 nm)/ mCBP (20 nm)/ mCBP:PO-T2T (20 nm)/ mCBP:PO-T2T:4.0 wt.% Ir(bt)_2_(acac) (2 nm)/ PO-T2T (40 nm)/LiF (0.8 nm)/Al; ITO/MoO3 (3 nm)/ mCBP (20 nm)/ mCBP:PO-T2T:4.0 wt.% Ir(bt)_2_(acac) (20 nm)/ PO-T2T (40 nm)/LiF (0.8 nm)/Al.


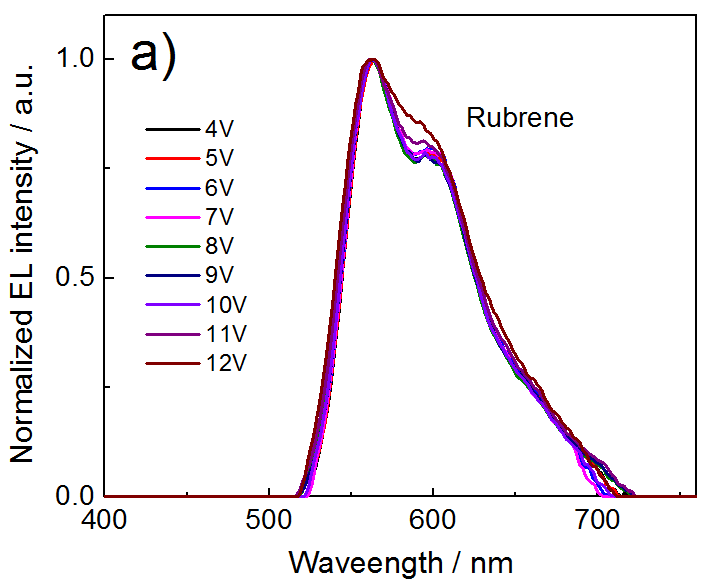

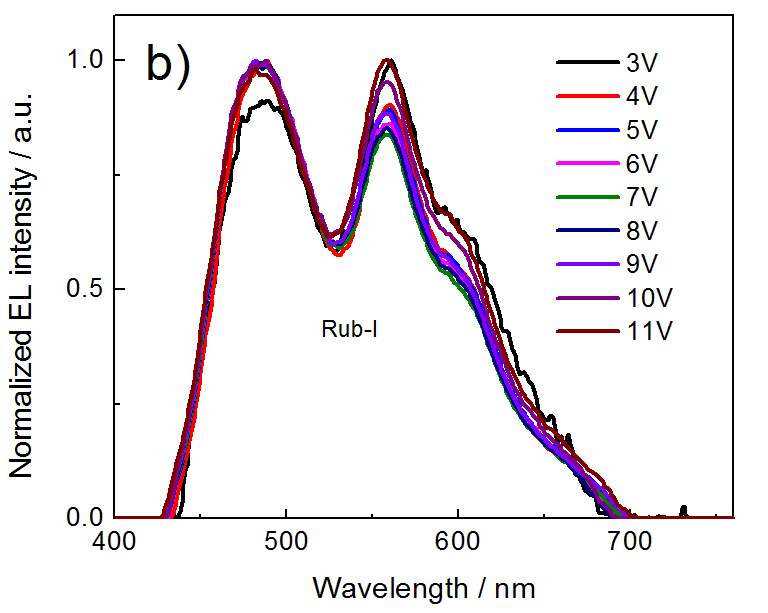

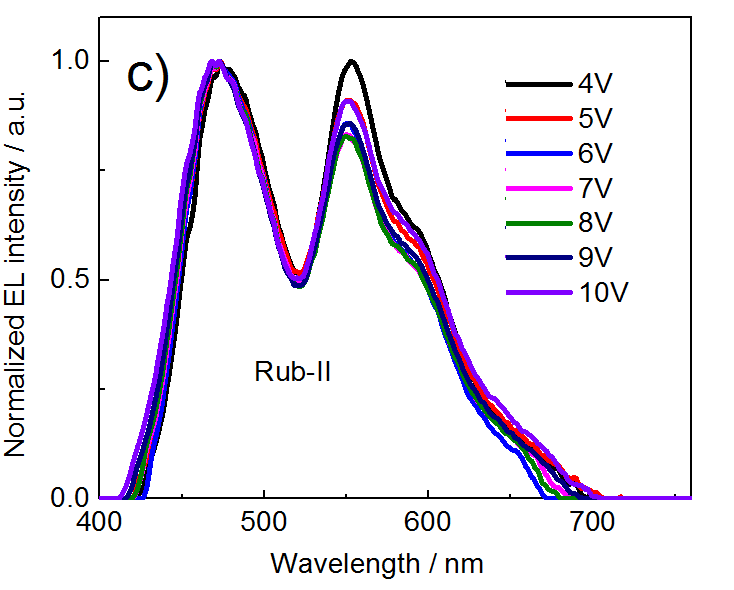

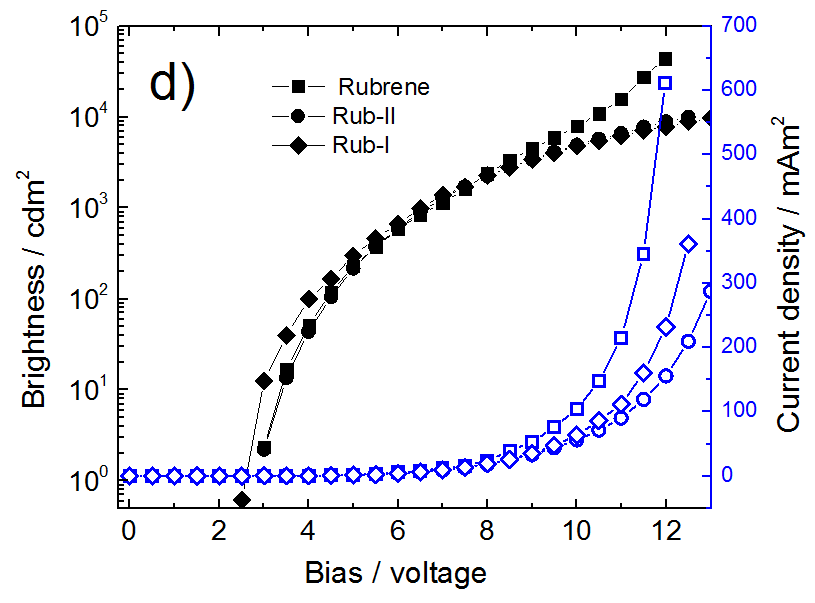

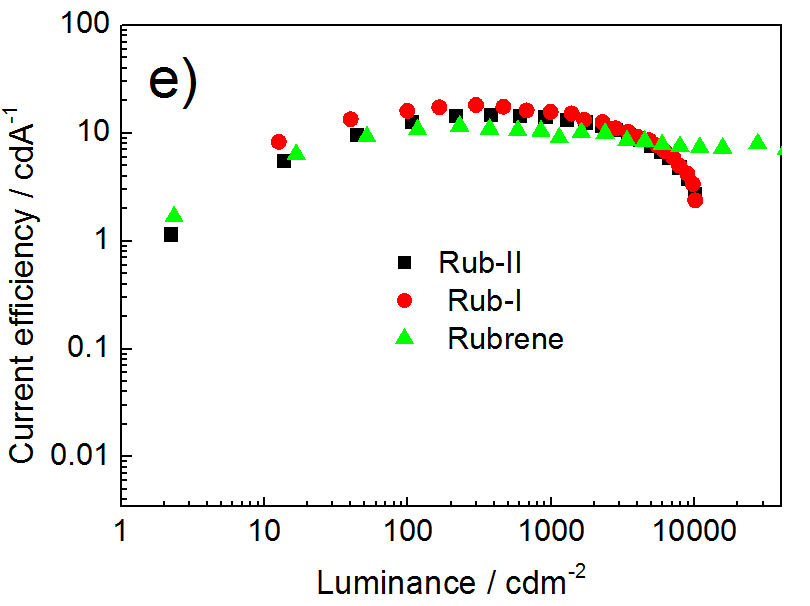


**Figure S5.** Normalized EL spectra of Rubrene doped a) orange device, b) one emitting layer and c) two emitting layer based WOLEDs. d) Current density and brightness and e) current efficiency of Rubrene doped orange device, one emitting layer and two emitting layer based WOLEDs. The device structures are: ITO/MoO3 (3 nm)/ mCBP (20 nm)/mCBP:PO-T2T:0.4 wt.% Rubrene (20 nm)/ PO-T2T (40 nm)/LiF (0.8 nm)/Al; ITO/MoO3 (3 nm)/ mCBP (20 nm)/ mCBP:PO-T2T (20 nm)/ mCBP:PO-T2T:1.0 wt.% Rubrene (5 nm)/ PO-T2T (40 nm)/LiF (0.8 nm)/Al; ITO/MoO3 (3 nm)/ mCBP (20 nm)/mCBP:PO-T2T:1.0 wt.% Rubrene (20 nm)/ PO-T2T (40 nm)/LiF (0.8 nm)/Al.


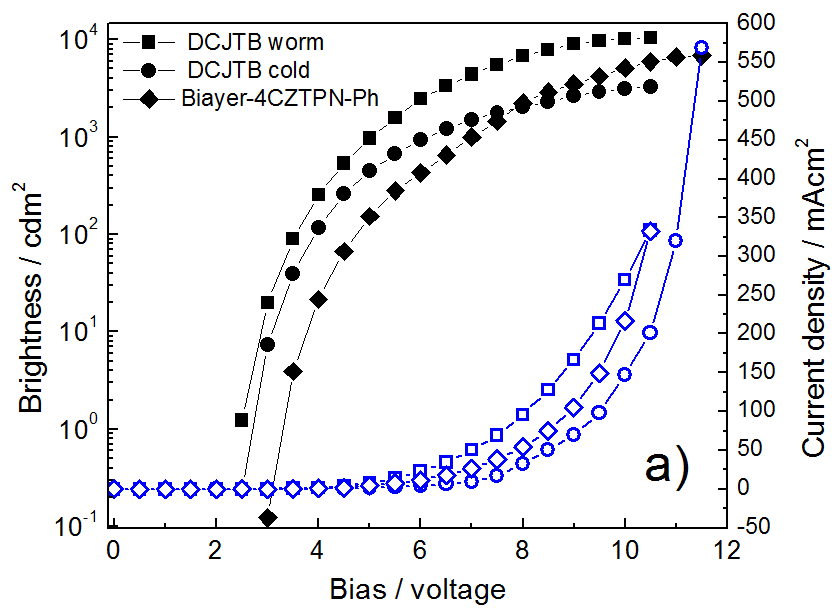

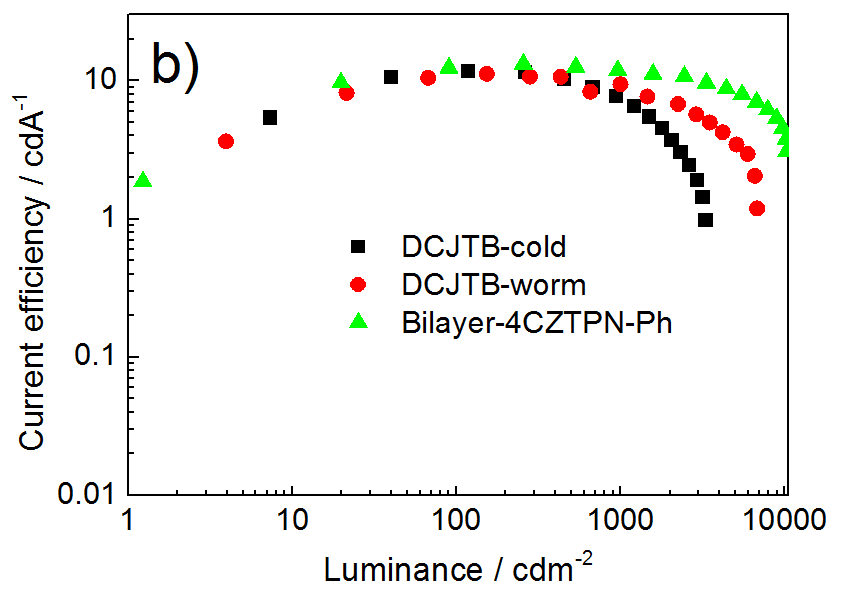


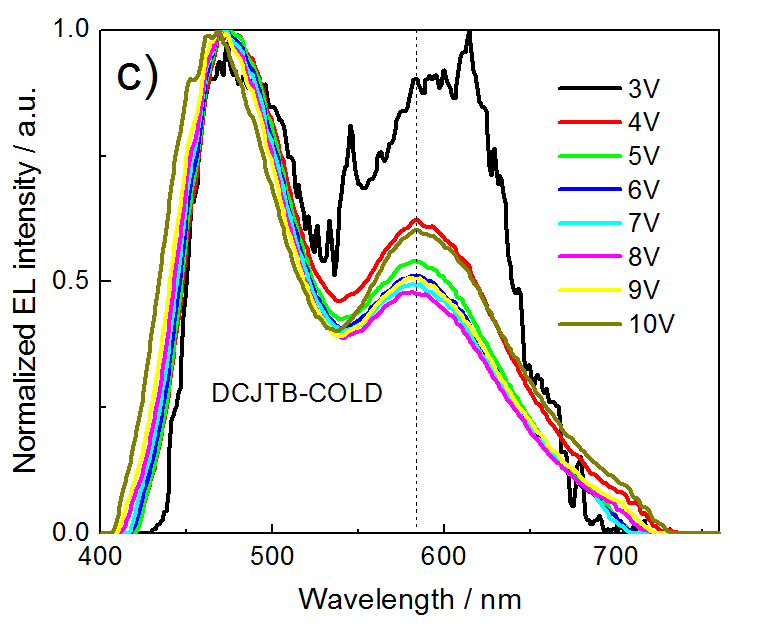

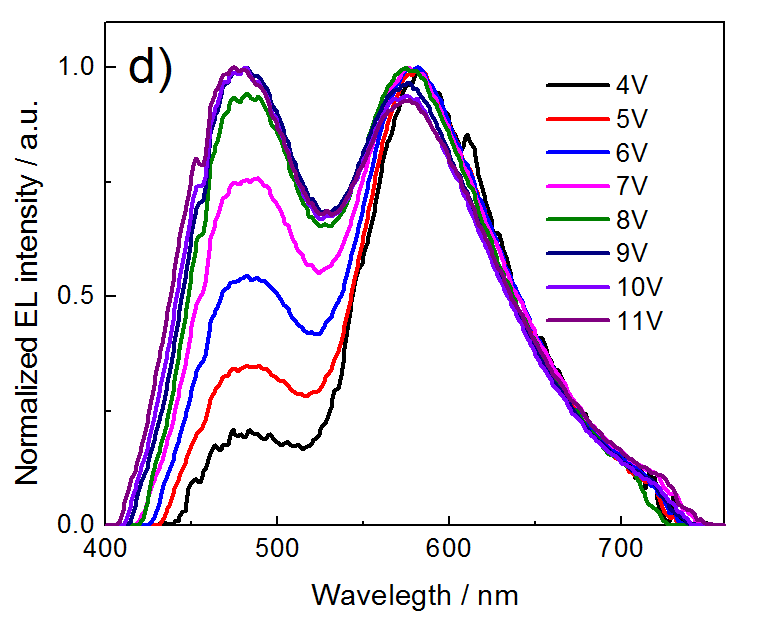


**Figure S6.** a) Luminance and current and b) current efficiency of DCJTB doped warm and cold WOLED as well as the 4CZTPN-Ph doped bilayer WOLED. b) EQE vs luminance of three devices. c) EL spectra of the DCJTB based warm WOLED at different bias from 3 V to 10 V. d) EL spectra of the 4CZTPN-Ph based WOLED at different bias from 4 V to 11 V. The device structures are: ITO/MoO3 (3 nm)/ mCBP (20 nm)/mCBP:PO-T2T:0.4 wt.% DCJTB (20 nm)/ PO-T2T (40 nm)/LiF (0.8 nm)/Al; ITO/MoO3 (3 nm)/ mCBP (20 nm)/mCBP:PO-T2T:0.2 wt.% DCJTB (20 nm)/ PO-T2T (40 nm)/LiF (0.8 nm)/Al; ITO/MoO3 (3 nm)/ mCBP (20 nm)/ mCBP:PO-T2T (20 nm)/ PO-T2T:5.0 wt.% 4CZTPN-Ph (10 nm)/ PO-T2T (40 nm)/LiF (0.8 nm)/Al.
